# Molecular characterization of coat color gene in Sahiwal versus Karan Fries bovine

**DOI:** 10.1186/s43141-021-00117-2

**Published:** 2021-01-29

**Authors:** Talla Sridhar Goud, Ramesh Chandra Upadhyay, Vijaya Bhaskar Reddy Pichili, Suneel Kumar Onteru, Kiranmai Chadipiralla

**Affiliations:** 1 Climate Resilient Live Stock Research Centre, ICAR-National Dairy Research Institute, Karnal, Haryana 132001 India; 2 Department of Biotechnology, Vikrama Simhapuri University, Andhrapradesh, Nellore, 524320 India; 3 Department of Life Science & Bioinformatics, Assam University Diphu Campus, Diphu, Assam 782462 India; 4 Molecular Endocrinology, Functional Genomics and Structural Biology, Animal Biochemistry Division, ICAR-National Dairy Research Institute, Karnal, Haryana 132001 India

**Keywords:** Coat color, *Melanocortin 1 receptor* gene, Karan Fries cattle (Bos taurus taurus), Sahiwal (Bos taurus indicus), SNPs

## Abstract

**Background:**

*Melanocortin-1-receptor* gene (*MC1R*) plays a significant role in signaling cascade of melanin production. In cattle, the coat colors, such as red and black, are an outcome of eumelanin and pheomelanin pigments, respectively. The coat colors have become critical factors in the animal selection process. This study is therefore aimed at the molecular characterization of reddish-brown coat-colored Sahiwal cattle in comparison to the black and white-colored Karan Fries.

**Results:**

The Sequence length of the *MC1R* gene was 954 base pairs in Sahiwal cattle. The sequences were examined and submitted to GenBank Acc.No. MG373575 to MG373605. Alignment of both (Sahiwal and Karan Fries) protein sequences by applying ClustalO multiple sequence alignment programs revealed 99.8–96.8% sequence similarity within the bovine. *MC1R* gene phylogenetic studies were analyzed by MEGA X. The gene *MC1R* tree, protein confines, and hereditary difference of cattle were derived from Ensemble Asia Cow Genome Browser 97. One unique single-nucleotide polymorphism (c.844C>A) (SNP) was distinguished. Single amino acid changes were detected in the seventh transmembrane structural helix region, with SNP at p.281 T>N of *MC1R* gene in Karan Fries cattle.

**Conclusions:**

In this current research, we first distinguished the genomic sequence of the *MC1R* gene regions that showed evidence of coat variation between Indian indigenous Sahiwal cattle breed correlated with crossbreed Karan Fries. These variations were found in the *Melanocortin 1 receptor* coding regions of the diverse SNPs. The conclusions of this research provide new insights into understanding the coat color variation in crossbreed compared to the Indian Sahiwal cattle.

**Graphical abstract:**

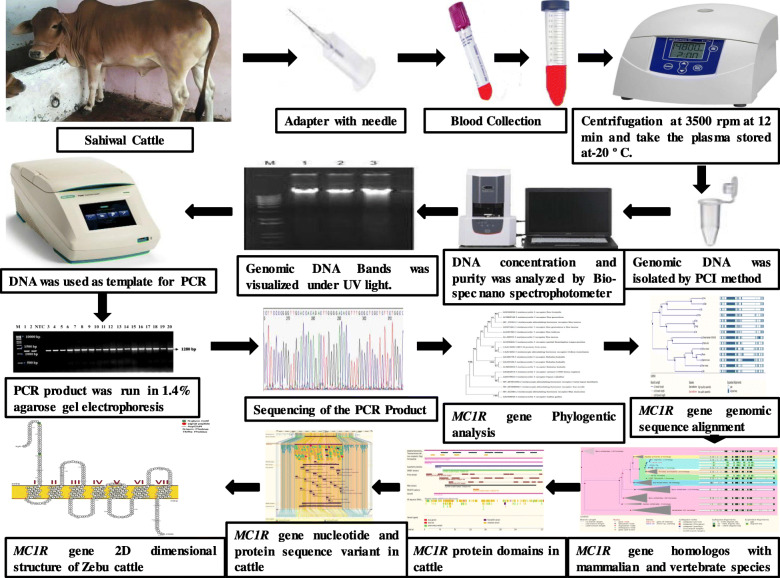

**Supplementary Information:**

The online version contains supplementary material available at 10.1186/s43141-021-00117-2.

## Background

Coat color is a part of the major essential features for identifying the modern breeds of cattle. Each breed has its own specific phenotypic, physiological, hormonal, and metabolic functions to sustain and adapt to diverse agro-geographical and tropical climatic conditions. Coloration is an effective part of variable phenotypical traits in a diversity of animals, and there are several hypotheses for its function, such as camouflage and signaling of diverse detectable progression [1–3]. In mammals, coat color has been associated with their production and environmental adaptation. Darwin for the first time stated that a broad range of domestic animals shares multiple phenotypic characteristics, the most evident of which is a wide variety of coat colors [4, 5]. In vertebrates, the only character other than variable coat colors that occur The Sequence length of the *MC1R* gene was 954 base pairs. Coat color phenotypes are useful in the identification of diverse cattle breeds and other livestock. The coat color in animals is dependent on the percentage of eumelanin coating for black-brown pattern [6] to that of the pheomelanin coating for yellow-reddish pattern [7]. The α-melanocyte-stimulating hormone (α-MSH) and the melanocortin-1 receptor (*MC1R*) have been implicated to execute a pivotal function in regulating the pigment synthesis in several animal species for coat coloration [8, 9]. The hormone α-MSH, upon binding with *MC1R* results in stimulating of adenylate cyclase through the G-protein coupled receptors, consequently leading to high intracellular cAMP levels. Tyrosinase is a well-known rate-limiting enzyme in the melanin pigment synthesis pathway, whose activity is dependent on the prominence of cyclic AMP [10–13]. Therefore, *MC1R* performs an essential role in the production of melanin in melanocytes. The variations within the *MC1R* gene must reveal an effect on the coat color of the diverse animal species like the cow, buffalo, Chinese yakow [14–18] sheep, goat [19, 20], pigs, horses [21, 22] foxes, dogs [23, 24], cats, and mice [25, 26].

The *MC1R* gene encoding for a 7-transmembrane domain [27] contains a single exon spanning over 954 base pairs. Its extension locus is positioned on chromosome 18 in cattle. It is well known that variation in the regulation and expression of the *MC1R* gene plays a vital role in causing variations in the pigmentation patterns [28]. For instance, differentiation in base coat color of the cattle is due to the hereditary alterations in the *MC1R* gene, traditionally termed as the extent locus, with alleles coding for black (ED), red (e), and wild-type (E+). Those alternative alleles were identified to observe a dominance model, in which ED > E+ > e. The native population allele (E+) encodes for a receptor that responds to both the α-melanocyte-stimulating hormone (α-MSH) ligand and its competitor, agouti-signaling protein (ASP). The effective ED allele causes a point mutation altering the amino acid from leucine to proline, and promoting the constitutive expression of an active receptor, which acts on most liable eumelanin because of its α-MSH ligand binding mimicry [29].

Sahiwal cattle is a milch breed that mainly existed in the home track of Punjab, Haryana, Uttar Pradesh, and Madhya Pradesh states of India. Its milk yield ranges from 1140 to 3180 kg per lactation. It is remarkably resistant to tick-borne infections, thermo-tolerant, and perceived for its enhanced protection to internal and external parasites. The color of these animals ranges from lighter reddish-brown to a thicker predominant red occasionally with varying degrees of white patches over the neck and underline. Further, the intensity of the color normally shades in the extremities such as legs, tail, and head. In the current research study, the *MCIR* gene was amplified from the genomic DNA of Sahiwal cattle breed and compared its sequence with that of Karan Fries breed. In addition, a small cohort of Sahiwal cattle was genotyped for a SNP of the *MC1R* gene, c.844C>A.

## Methods

### Experimental environment and geographical location

All the experimental cows were housed in Climate Resilient Livestock Research Centre (CRLRC) located in Karnal district, Haryana state, India. The region is positioned at an altitude of 250 ms elevated than the mean sea level (MSL) geographically located at 29° 42″ N 79° 54″ E, latitude, and longitudinal view, respectively. The location experiences a huge variation in the temperatures measured between summers and winters. While the summers record a maximum temperature of about 46 °C, winter temperature drops to as low as 0 °C. The mean rainfall from July to August month is around 700 mm.

### Experimental design

A total of 30 clinically healthy Sahiwal cattle free from any physical abnormalities were selected in this study. All animals were closely scrutinized and maintained. Blood samples (5–7 ml) were collected from the animals by jugular vein perforation with the help of 18 G needle in K2-EDTA (1 mg/ml concentration) anticoagulant Vacutainer tubes® (BD Biosciences USA) under sterile conditions. The coat colors of the animals were determined by direct visual inspection and recorded at the time of blood specimen collection. Blood samples isolated were cautiously stored at − 20 °C for further molecular studies.

### Genomic DNA isolation from whole blood samples

The genomic DNA was isolated by standard proteinase K digestion process and phenol, chloroform, and isoamyl alcohol method [30]. To illustrate the quality of the isolated DNA, 200 ng of genomic DNA was loaded on 0.8% agarose gel electrophoresis along with 1 kb marker. Genomic DNA quality and intensity were also investigated by Nanodrop (Shimadzu BioSpec-nano, Japan). We achieved the DNA sample quality ranging between 1.80 and 1.85. An amount of 50–100 ng/μl of genomic DNA was applied as the template for the polymerase chain reaction.

### Polymerase chain reaction (PCR)

The primer sequences were designed using the reference gene sequence of *Bos taurus* cattle

(GenBank accession number: NM_174108) and the Primer 3.0 software (Applied Biosystems, USA). To amplify the *MC1R* sequence of 1280 bp, we employed the following primers set.

Forward Primer: 5′-GGACCCTAGGAGAGCAAGCAC-3′,

Reverse Primer: 5′-CTCACCTTCGAGGATGGTCTA-3′.

**Fig. 1:**
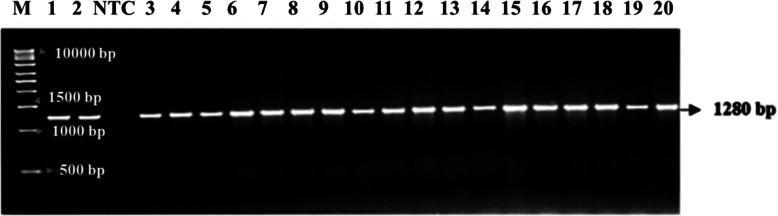
Sahiwal Cattle *MC1R* gene PCR product amplication (M) marker 1kb (1-20) number of animals

The PCR was performed in a reaction volume of 25 μl, which contained 12.5 μl 2X PCR Master mix buffer (Sigma, USA), 1 μl forward primer (10 pico-moles/μl), 1 μl reverse primer (10 pico-moles/μl), 2 μl of DNA (50–100 ng), and 8.5 μl of nuclease-free water. The PCR protocol parameters and the procedure is as follows: 95 °C for 3 min; 35 cycles at 94 °C for 1 min, 59 °C for min, and 72 °C for 1 min; and final extension cycle at 72 °C for 10 min (Applied BioSystem Gene Amp® PCR 9700 USA). Each PCR product was separated by 1.4% agarose gel along with 1 kb molecular ladder (Sigma, USA). After cautious staining of the gel with ethidium bromide dye (0.5 μg/ml), amplified bands were observed. Band pictures were clicked and stored by Gel documentation imaging equipment (Bio-Rad, USA) (Fig. 1).

### *MC1R* gene PCR product sequencing and analysis

A complete quantity of 50 μl PCR products was sequenced proceeding with 10 μl of the forward and reverse primers of the *MC1R* gene. All the PCR products were washed in the spin column kit (Sigma, USA) with the maintenance of the ABI3730 DNA analyzer (Applied Biosystems) sequencing service done by Sci-Genom, Kerala, India, Pvt. Ltd. The sequence chromatogram ABI files were visualized and analyzed by using Bio-Edit version 7.2. The *MC1R* gene Open reading frames were distinguished by using the ORF finder tool by NCBI (http://www.ncbi.nlm.nih.gov/orffinder/) and the nucleotide sequence was interpreted using the translate tool by ExPASy (http://www.expasy.org). The *MC1R* protein signal peptide prediction was recognized and exhibited by applying SignalP 4.0 server online (http://www.cbs.dtu.dk/services/SignalP/) based on the neural interface instructed on eukaryotes [31]. The bio-physicochemical properties of *MC1R* proteins were analyzed by applying Protparam server ExPASy [32]. The integrated membrane proteins were analyzed by PROTTER online server (http://wlab.ethz.ch/protter) [33]. *MC1R* amino acid sequence, secondary structure, and hydrophobic surface accessibility (see Supplementary file) were predicted by NetSurfP-2.0 webserver (http://www.cbs.dtu.dk/services/NetSurfP/) [34]. The distinguished *MC1R* protein sequences were associated with the protein data sequence of *Bos frontalis*, *Bos grunniens*, *Bos taurus*, and *Bubalus bubalis* available at NCBI GenBank database using the pBLAST program.

### Phylogenetic analysis

The *MC1R* protein sequences of diverse mammalian varieties were regained from the NCBI GenBank database. The phylogenetic tree study was done by MEGA X software by applying the Maximum Parsimony method. This method takes a minimum basic number of mutations into consideration to analyze and elucidate any given series of sequences that are positioned together and does not allow any modification or amendment at every single site. Using this method, the evolutionary records were obtained from all the parsimony sites represented in the parenthesis, in which the rate of duplicate trees, the associated grouped taxa from the bootstrap, distributed (1000 replicates) is presented near to the branches. Limited than fifty percentage of bootstrap replicates are deflated but the branches following to partitions were reproduced [35]. A total of nine amino acid sequences were carried in the study. In the last dataset, a total of 317 positions were covered. The molecular genetic analyses were carried in MEGA X [36].

## Results

The *MC1R* gene was amplified and sequenced for the first time in Indian zebu Sahiwal cattle breed. The gene sequence fragment was achieved to be about 1280 base pairs that comprises coding regions and parts of the 5′ and 3′ noncoding regions (UTR) of the *MC1R* gene (185 and 141 bp sequentially). The complete coding domain of the Sahiwal cattle *MC1R* gene ORF length is 954 base pairs long and it encodes a protein of 317 amino acids. This *MC1R* gene is related with a specific G-protein linked receptor (GPCR). *MC1R* gene associated with the *B. indicus* reference gene sequences showed 100% similarity. The *MC1R* gene sequence was submitted to the NCBI GenBank database with accession number MG373575 to MG373605. Further, the *MC1R* gene sequence was translated by using Expaxy protein translational tool. The *MC1R* protein sequence was further characterized as described below.

### *MC1R* protein sequence physicochemical characteristics in the ruminant species

The entire coding region of cattle *MC1R* protein (GenBank accession number NM_174108) was compared with Sahiwal (*Bos indicus*), gayal (*Bos frontalis*), yak (*Bos grunniens*), Karan Fries (*Bos taurus*) cattle, buffalo (*Bubalus bubalis*), sheep (*Ovis aries*), goat (*Capra hircus*), and horse (*Equus caballus*). The *MC1R* (954 bp) conceal a protein of 317 amino acids with a predicted molecular mass of 34,937.00, 34,935.02, 34,946.00, 34,946.05, 34,817.88, 34,918.95, 34,859.89, and 35,213.36 daltons and iso-electric point of the protein was pI = 8.97, 8.97, 8.97, 9.05, 8.97, 8.97, 8.97, and 8.69 sequentially in the order of species mentioned above. The average number of amino acids in proportions for all the nine species is shown in Table 1. The other physicochemical characteristics of the *MC1R* protein sequence of the other mammals are presented in Table 2. Like protein, motif predictions were also made by employing the PROSITE motif exploration tool and other transmembrane helix predictions were investigated by using transmembrane prediction tools. Overall, we observed the 7-transmembrane helix region including inside and outside regions. A total of 317 amino acids with the 7-transmembrane structural helix region was identified in the *MC1R* protein. The *MC1R* protein sequence with other mammals was characterized by further studies.

**Table 1:** *MC1R* protein amino acid frequency sites used in the mammalian species all frequencies are given in percent (%)

Name of the Species	Ala	Cys	Asp	Glu	Phe	Gly	His	Ile	Lys	Leu	Met	Asn	Pro	Gln	Arg	Ser	Thr	Val	Trp	Tyr	Total
*B. indicus*	9.78	4.42	1.89	1.89	5.36	5.05	2.52	7.57	1.89	17.35	2.21	3.15	4.42	4.10	4.73	5.99	4.42	9.15	1.26	2.84	317
*B. frontalis*	9.46	4.42	1.89	1.89	5.05	5.05	2.52	7.57	1.89	17.67	2.21	3.47	4.42	4.10	4.73	5.99	4.42	9.15	1.26	2.84	317
*B. grunniens*	9.78	4.42	1.89	1.89	5.05	5.05	2.52	7.57	1.89	17.67	2.21	3.47	4.42	4.10	4.73	5.99	4.10	9.15	1.26	2.84	317
*B. taurus*	9.46	4.42	1.58	1.89	4.73	5.05	2.52	7.57	1.89	17.35	2.21	3.79	4.73	4.10	5.05	5.99	4.42	9.15	1.26	2.84	317
*B. bubalis*	9.78	4.42	1.89	1.89	4.73	4.73	2.52	7.26	1.89	17.67	2.21	3.47	4.42	4.10	4.73	6.62	4.10	9.46	1.26	2.84	317
*C. hircus*	9.78	4.42	1.89	1.89	4.73	4.73	2.52	7.26	1.58	17.35	2.52	3.47	4.73	3.47	5.05	6.62	4.42	9.46	1.26	2.84	317
*O. aries*	9.46	4.42	1.89	1.89	4.73	4.73	2.52	7.26	1.58	17.35	2.52	3.47	4.42	3.79	5.05	6.62	4.42	9.78	1.26	2.84	317
*O. moschatus*	9.78	4.42	1.89	1.89	4.73	4.73	2.52	7.26	1.58	17.35	2.52	3.47	4.73	3.47	5.05	6.62	4.42	9.46	1.26	2.84	317
*E. caballus*	7.57	4.10	1.89	2.21	5.05	4.73	3.15	6.62	1.89	18.93	3.15	2.84	4.42	3.79	4.10	7.26	4.73	9.15	1.26	3.15	317
**Avg. %**	**9.43**	**4.38**	**1.86**	**1.93**	**4.91**	**4.87**	**2.59**	**7.33**	**1.79**	**17.63**	**2.42**	**3.40**	**4.52**	**3.89**	**4.80**	**6.41**	**4.38**	**9.32**	**1.26**	**2.87**	**317**

*Ala* alanine, *Asp* aspartic acid, *Cys* cysteine, *Glu* glutamic acid, *Phe* phenyl alanine, *Gly* glycine, *His* histidine, *Ile* iso-leucine, *Lys* lysine, *Leu* leucine, *Met* methionine, *Asn* asparagine, *Pro* proline, *Gln* glutamine, *Arg* arginine, *Ser* serine, *Thr* theronine, *Val* valine, *Trp* tryptophan, *Tyr* tyrosine

**Table 2:** Physicochemical properties of *MC1R* protein sequence of the mammalian species

S.NO	Species	Open reading frame length in base pairs	Amino acids length	Chromosome number	NCBI accession number	% Identity with ***Bos indicus***		Negatively charged residues (Asp + Glu)	Positively charged residues (Lys + Arg)	N-Glyc sites	O-Glyc sites	Phosphorylation sites
						Nucleotide	Amino acid					
1	*B. indicus*	954	317	18	MG373575	–	–	12	21	6	4	21
2	*B. frontalis*	954	317	18	HM488960	99.9	99.68	12	21	6	4	21
3	*B. grunnesis*	954	317	18	FJ624478	99.79	99.37	12	22	6	4	21
4	*B. taurus*	954	317	18	NC037345	99.2	99.37	11	22	6	2	21
5	*B. bubalis*	954	317	18	NW020228700	97.49	97.16	12	21	7	4	25
6	*C. hircus*	954	317	18	NW017189504	96.33	96.3	12	21	2	3	21
7	*O. aries*	954	317	14	NC019471	95.70	96.85	12	21	2	0	21
8	*O. moschats*	954	317	18	Y13958	96.33	96.85	12	21	6	3	21
9	*E. caballus*	954	317	3	NC009146	85.94	83.91	13	19	7	2	21

### Comparative investigation of the *MC1R* protein sequence with other mammals

**Fig. 2:**
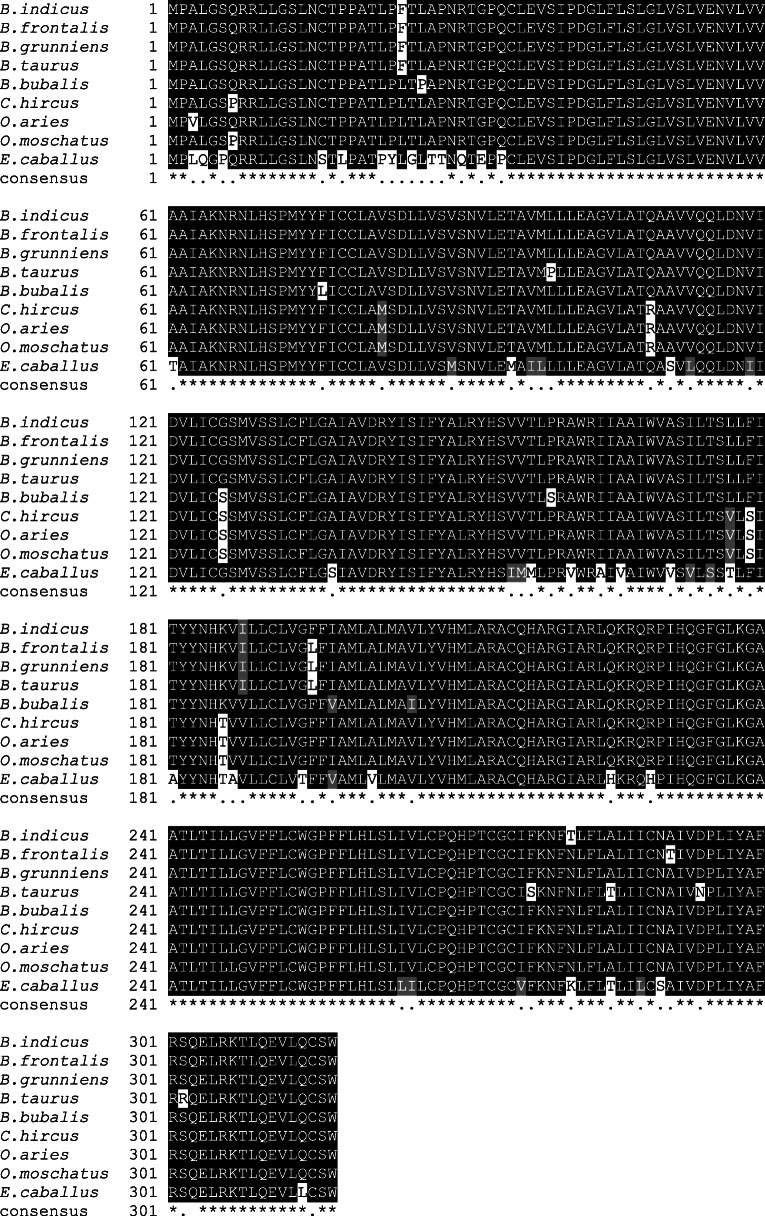
Clustal Omega multiple sequence alignment by MUSCLE (3.8). Alignment of amino acid sequences of *MC1R* gene *B. indicus* (MG373575), *B. frontalis* (HM488960), *B. grunniens* (FJ624478), *B. taurus* (NC037345), *B. bubalis* (NW020228700), *C. hircus* (NW017189504), *O. aries* (NC019471), *O. moschats* (Y13958), *E. caballus* (NC009146), illustrates the high identity. The black color region was highly correlated to other mammalian species * a star mark designates a conserved amino acid sequence of the *MC1R* protein among the all mammalian species

We compared the *MC1R* protein sequences of the Sahiwal, gayal, wild yak, Karan Fries, buffalo, goat, sheep, and horse. The results showed a high homology identity. Amino acid sequence alignment of the *MC1R* protein was done by using multiple sequence alignment Clustal Omega, (MUSCLE 3.8 version), European Bioinformatics Institute. The *MC1R* amino acid sequence showed very high homology with other mammals as compared with *Bos frontalis* 99.68%, *Bos grunniens* 99.37%, *Bos taurus* 99.37%, *Bubalus bubalis* 97.13%, *Capra hircus* 96.85%, *Ovis aries* 96.85.%, and *Equus caballus* 83.91% (Fig. 2).

### Comparative molecular phylogenetic analysis of the Sahiwal *MC1R* with other mammals

**Fig. 3:**
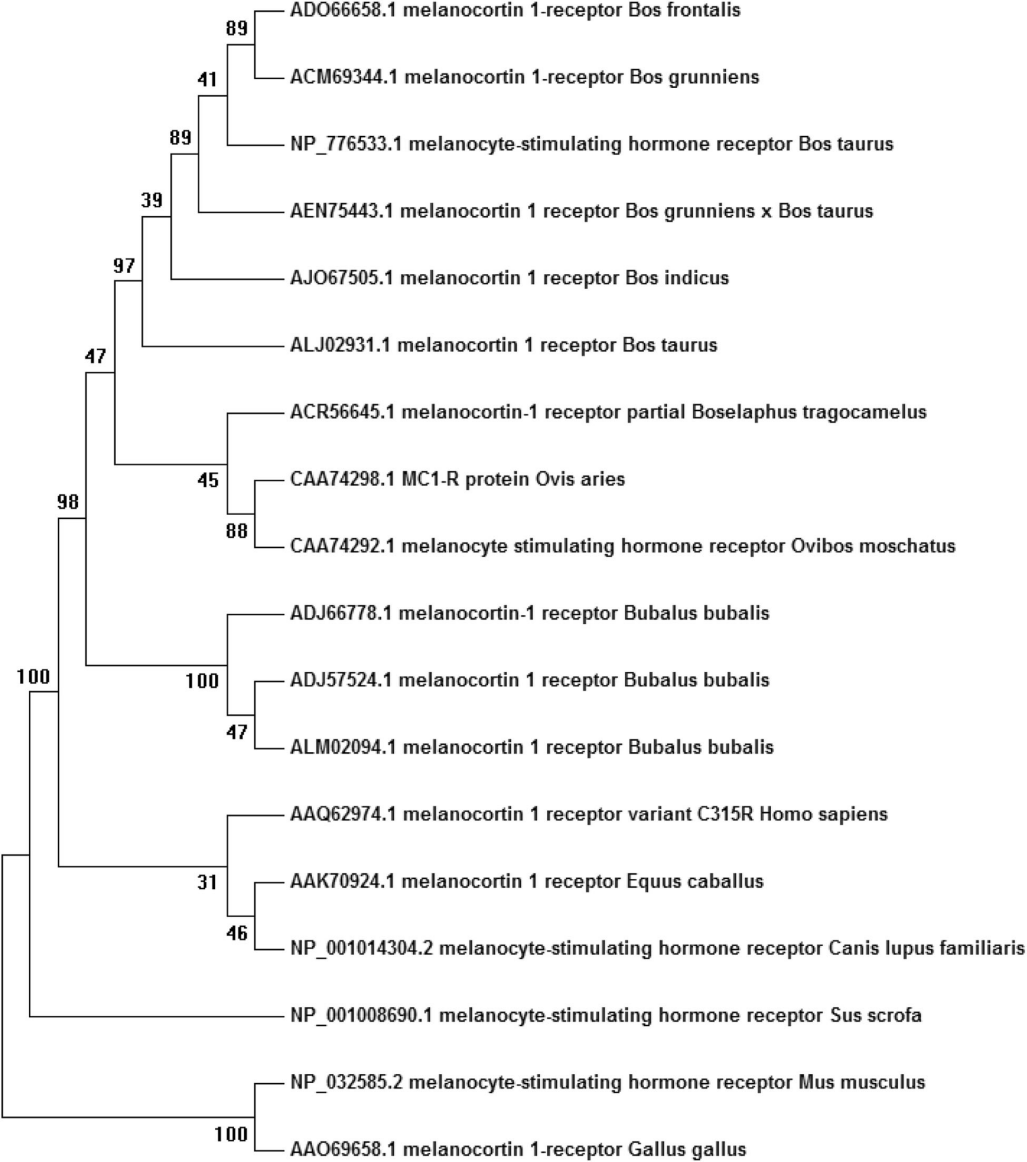
Phylogenetic tree reviews based on amino acid arrays of the *MC1R* protein of Sahiwal (*B. indicus*). The tree was assembled by the Maximum Parsimony scheme using MEGA X (Molecular Evolutionary Genetics Analysis) software with bootstrap rates estimated for 1000 replicates

After determining the amino acid alignment sequence of *MC1R*, we analyzed the *MC1R* protein sequence of the Sahiwal cattle breed. The phylogenetic tree was built based on the *MC1R* sequence of distinct mammalian ruminant species retrieved from GenBank to investigate the evolutionary relationship along with the Sahiwal cattle and *MC1R*s of other ruminant species. The results indicated that *MC1R* of the Sahiwal cattle (*Bos indicus*) have shown maximum close relations with yak (*Bos frontalis*), Gayal (*Bos grunniens*), Karan Fries (*Bos taurus*), and *Bubalus bubalis*, and a modest relation was observed with other ruminant species such as *Capra hircus*, *Ovis aries*. The sequence integrity was perceived to be least when related with *Homo sapiens* and *Canis lupus familiars*, *Equus caballus*. The *MC1R* sequence of Sahiwal revealed 100% relation with that of *Mus musculus, Gallus gallus*, and *Sus scrofa* (Fig. 3).

**Fig. 4:**
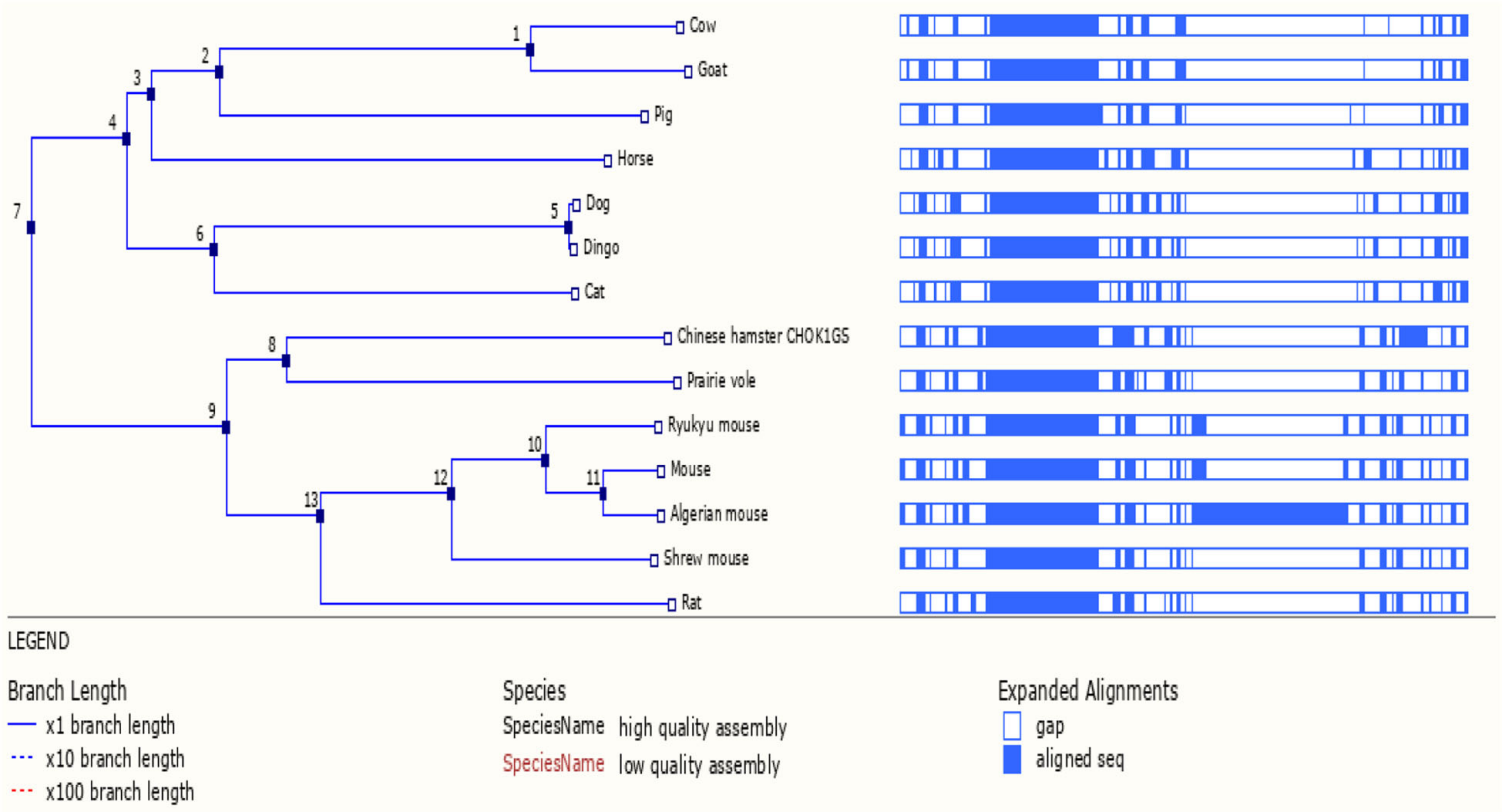
*MC1R* gene genomic sequence alignment in different vertebrates species

The *MC1R* gene genomic region alignment was related with the other thirteen mammalian species (Fig. 4). Among them, the first internode represents genomic sequence alignment of the cattle and goat that are closely related to the same taxa. The second, third, fourth, fifth, and sixth branch internodes were connected with the pig, horse, dog, and dingo, followed by the seventh branch with cat, respectively. Eight and ninth branches were interconnected with the Chinese hamster and Prairie vole, followed by the ten, eleven, twelve, and thirteen internodes, linked with Ryukyu mouse, mouse, Algerian mouse, Shrew mouse, and rat.

**Fig. 5:**
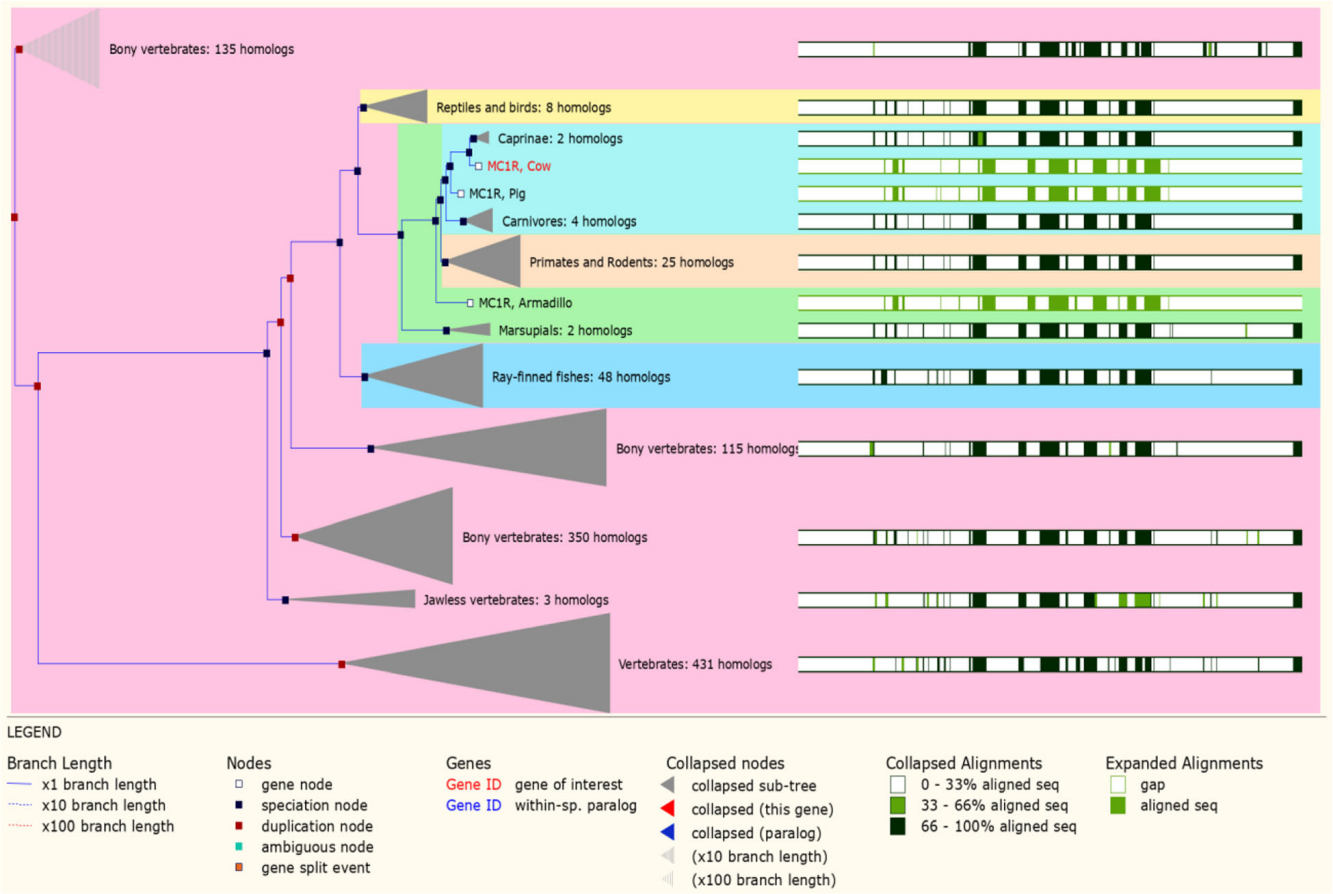
*MC1R* gene tree homologs in different mammalian and vertebrates

Moreover, the gene *MC1R* tree of cow was compared with vertebrate species by using Ensemble Asia cow genome browser and we retrieved the *MC1R* reference sequence (ENSBTAT00000032494) (Fig. 5) that was highly homologous with other vertebrates and mammalian species. The major portion of *MC1R* gene sequence alignment was highly homologous in 431, vertebrate species, 350, 115 bony vertebrates’ and the gene sequence of the alignment was determined to be 100% similar. Among the primates and rodents, 25 different species revealed a very large homologous sequence. Moreover, 48 species of ray-finned fishes were remarkably homologous displaying 100% identity. The reptiles, birds, carnivores, jawless vertebrates, marsupials, and caprine species showing 100% identical homologous sequence was found to be 8, 4, 3, 2, 2. Cattle, pig, and armadillo sequence revealed 33–66% relevant homologs in their association. Subsequently, cattle and goat displayed the closely linked homologous sequence and connected to the identical branch node. Moreover, the *MC1R* protein region functions were defined below.

**Fig. 6:**
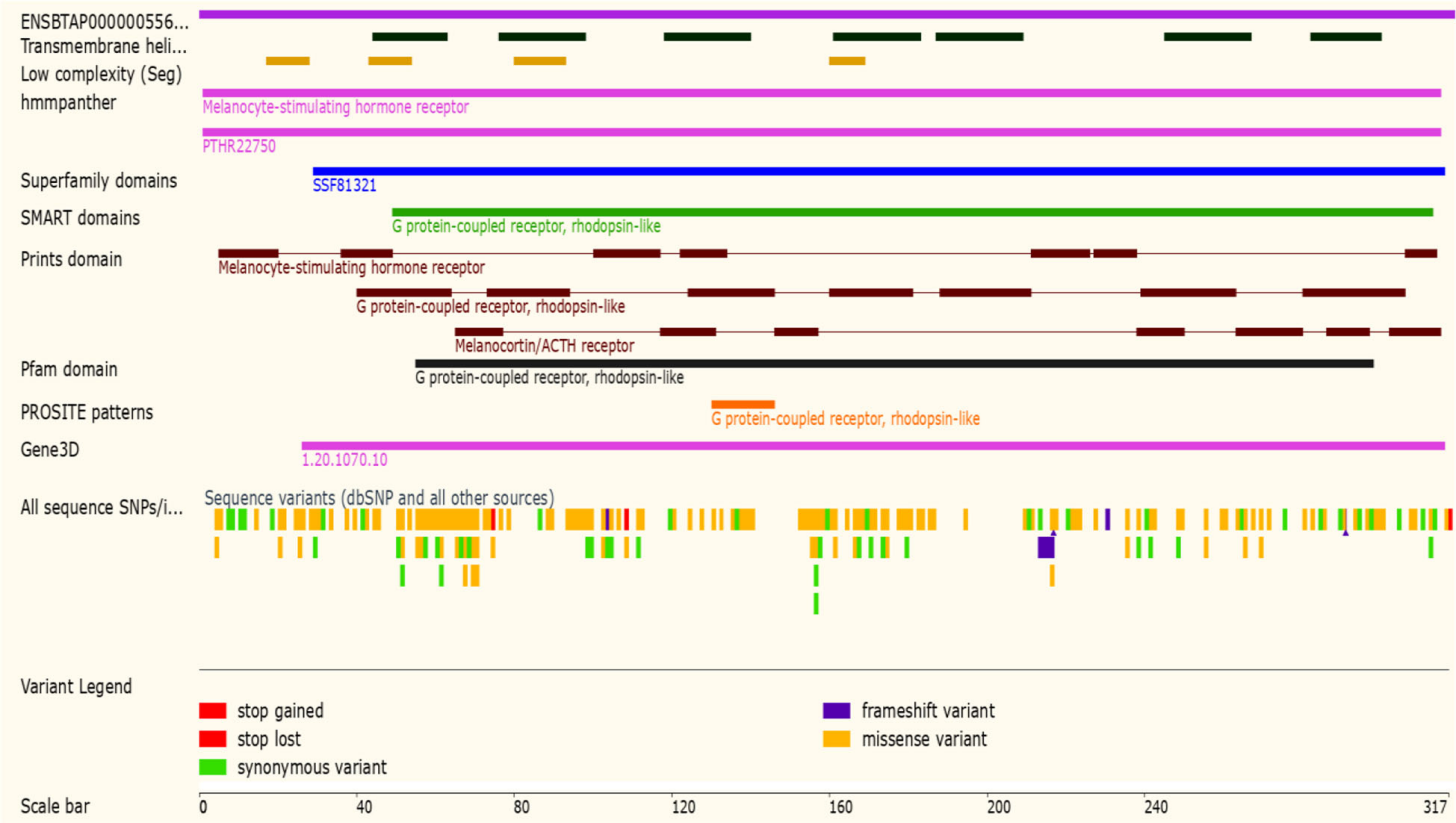
*MC1R* gene protein domains of the cattle

The *MC1R* protein region of the cattle is shown in Fig. 6. *MC1R* has seven transmembrane regions. *MC1R* gene codes the 317 amino acids; the *MC1R* protein domain was linked to a melanocyte-stimulating hormone receptor, adrenocorticotropic hormone receptor, and G-protein linked receptor, rhodopsin-like receptors. Protein super family domain region of the *MC1R* is linked with the G-protein linked receptor, rhodopsin-like receptor, serpentine-like receptor, seven transmembrane G-protein receptor, and chemoreceptors. PROSITE family was similar to the GPCR, rhodopsin-like 7TM region. In Fig. 6, the *MC1R* coding domain (shown in the red-shaded area) predominantly represents missense variation and those shown in the green-shaded area represent synonymous variants. In the coding region, the amino acids present at 75th and 109th, position showed loss of a function of the stop codon. Similarly, the amino acids present at 104 and 231 positions of the coding region showed four frame shift variations.

**Fig. 7:**
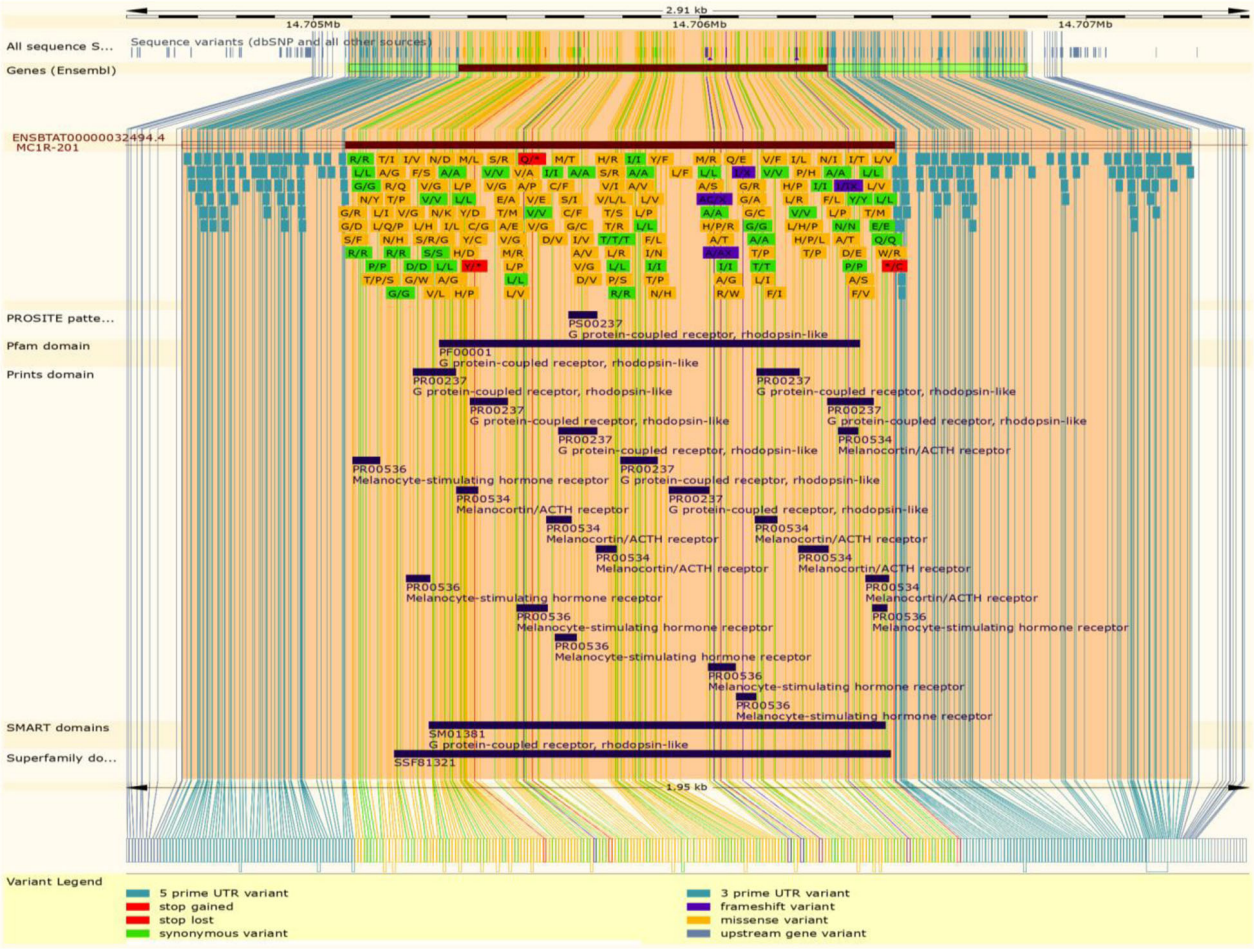
*MC1R* gene genomic and protein variations in cattle

The genetic variations in the genomic region of the *MC1R* gene are shown in Fig. 7. The full length of the *MC1R* gene which comprises the 1751 base pairs consists of 5′, 3′ flanking and un-translated regions; the range of the *MC1R* coding region was 954 base pairs. Most of the variants presented at the coding region and dominantly all were missense variants. Synonymous variants showed lightly in the coding domain. There were less frame shift variations found in the coding region of the *MC1R* gene in cattle.

### Identification of mutation in the *melanocortin 1 receptor* 7-transmembrane region

**Fig. 8:**
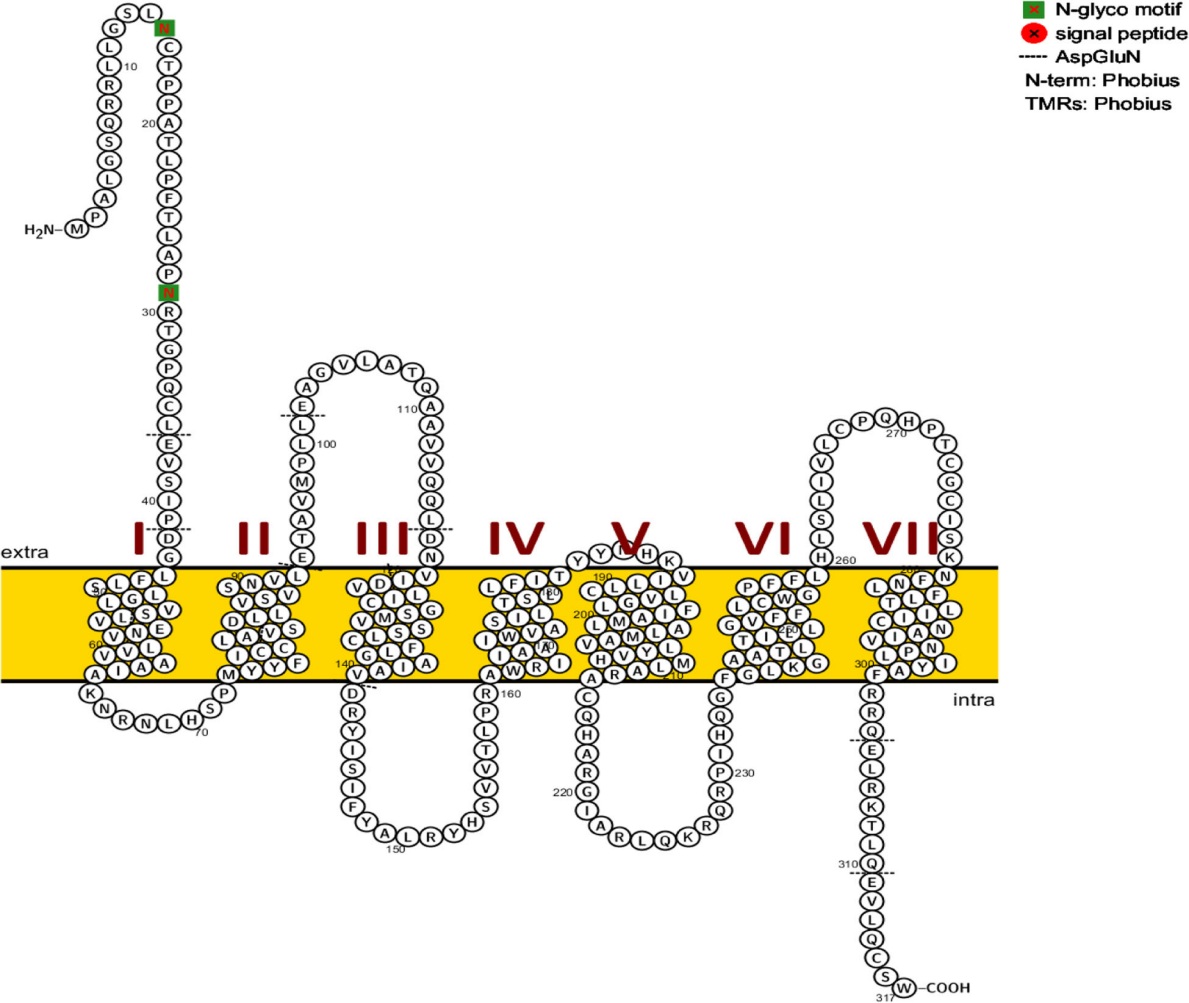
*MC1R* gene 2-Dimensional structure in cattle

Upon comparison of the Karan Fries with that of the zebu Sahiwal cattle, only one inter-breed SNP was identified (c.844C>A) in exonic region of the *MC1R* gene. One amino acid differences were found in the seven trans-membrane helix region of the *MC1R* protein between Sahiwal and Karan Fries (Fig. 8). The amino-terminal sequence of the *MC1R* protein contains a total of forty-three amino acids (1–43) (MPALGSQRRLLGSLNCTPPATLPFTLAPNRTGPQCLEVSIPDG). The (TM1) first trans-membrane structural helix precinct of the 7-TM comprises twenty-one amino acids (44–63) (LFLSLGLVSLVENVLVVAAIA) followed by the first intracellular domain region that contains eleven amino acids (64–75) (KNRNLHSPMYY). The (TM2) second transmembrane helix region contains twenty-three amino acids (76–98), (FICCLAVSDLLVSVSNVLETAVM) followed by the second extracellular domain region that contains nineteen amino acids (99–117) (PLLEAGVLATQAAVVQQLD), with an amino acid mutation occurring at (p.99 L>P) position. The (TM3) third trans-membrane structural helix region contains twenty-three amino acids (118–140) (NVIDVLICGSMVSSLCFLGAIAV) followed by the second intracellular domain regions containing nineteen amino acids (141–160) (DRYISIFYALRYHSVVTLP). The (TM4) fourth transmembrane structural helix contains twenty-two amino acids (161–183) (RAWRIIAAIWVASILTSLLFIT). Third extracellular transmembrane domain region contains two amino acids (184–186) (YY). Fifth (TM5) transmembrane structural helix region contains twenty-two amino acids presents (187–209), (NHKVILLCLVGLFIAMLALMAV). The third intracellular transmembrane domain region contains thirty-four amino (210–244) (LYVHMLARACQHARGIARLQKRQRPIHQGFGLKG) followed by the sixth (TM6) trans-membrane structural helix region that carries twenty-two amino acids (245–267) (AATLTILLGVFFLCWGPFFLHL). The fourth extracellular transmembrane domain region contains thirteen amino acids (268–281) (SLIVLCPQHPTCG), in which the p.281 T>N is the variation between Sahiwal and Karan Fries. Seventh (TM7) transmembrane structural helix region contains the eighteen amino acids (282–300) (CISKNFNLFLTLIICNAI) in position. The fourth intracellular transmembrane domain region contains sixteen amino acids (301–317) (RQELRKTLQEVLQCSW).

## Discussion

Coat color shows various types of shades in cattle and other mammalian species. *MC1R* gene plays a key role in regulating the eumelanin and pheomelanin biosynthesis pathways in mammals. Prior to the current study, there was no information available regarding coat color genes in Sahiwal (*Bos indicus*). Due to an inherited variation in the *MC1R* gene, which codes for the melanocyte-stimulating hormone (α-MSH) receptor, has been proved to be associated with the coat color of cows [9, 37, 38]. The *Bos* species *MC1R* has been broadly studied at three important loci such as the ED (dominant, dominant black), E+ (intermediate, recessive black), and e (recessive, red). The interchange of a T base (ED) with that of the C base (E+) at the 296th coding region leads to the variations of the amino acid from base T296C: p. Leu 99 Pro. The e allele derived from the deletion of a G at region 310/311 of the coding sequence. With regard to the skin color phenotype of Norwegian and Icelandic cattle herds, those with the ED allele present a dominant black color. Cattle possessing the e/e genotype always exhibited red, while animals homozygous or heterozygous for the wild-type allele E+/E+ and E+/e could produce either black, brownish, or red [14]. Red coat coloration is connected with the e locus of Holstein cow [15]. In Korean and Japanese beef cattle ED, *MC1R* gene has been briefly described in the e loci [39]. Black coat color Japanese cow had no homozygous allele’s e/e, and consequently obtained consistent, expressing their black coat color as ED and E+ alleles, which resulted in black pigmentation. In addition, the majority of Korean Hanwoo cattle varieties, which had a coat color changing from yellowish-brown to dark brown, including red skin color, were all homozygous for e/e [40]. Despite, Korean cattle owning a yellowish-brown color were perceived to have gene regularities of the E+/e (0.05) and e/e (0.95) [41]. In (*Bubalus bubalis*) black coat color of 49 buffaloes, white and grey coat color of 136 swamp buffaloes, 31 hybrid off-springs of river buffalo, and swamp buffaloes, *MC1R* gene was sequenced. Among these, a total of three SNPs were found. First SNP, c.618 G>C was the same sense mutation and among other SNPs, two were missense mutation c.310G>A and c.384C>T due to differences in the amino acid array p.S104G and p.I128M mentioned already. The black coat color was linked with the SNP104 in buffaloes [17]. In Thai white zebu (*Bos indicus*) cattle four SNPs c.296 T>C, c.416C >T, c.663A>C, and c.725A>C were reported earlier by Mekchay S [42]. Two novel alleles were already reported earlier in the Ed1 (c.T667C) (p.W223R) and Ed2 (c.651In12: p.218InARGI) in Brown Swiss cattle. In Simmental cattle, one of the allele ef was found (c.C890T), (p.T297I) by [43]. Recent studies in gayal *(Bos frontalis*) revealed nine SNPs including five SNPs in the coding domain (C201T, C583T, T663C, A871G, and T876C) and four SNPs (G-1A, C-106 T, A-127C, T-129C) in specific 5′ noncoding region published by previously [44]. Eight SNPs in Karan Fries crossbreed cattle were identified (c.296 T>C, c.583C>T, c.663 T>C, c.830C>A, c853G>A, c.880G>A, c.906C>G, and c.927C>T) recently in *MC1R* gene while compared with Tharparkar [16] along with we found the novel SNP (c.844C>A and p.281 T>N) in the present study*.* Hanna et al. reported both SNPs in *Bos indicus* source cattle at c.583C>T and c.663 T>C of *MC1R* [45]. Those were the only extra coding modifications that we recognized in this intron-less gene and they were in the complete phase with the *Bos indicus* E+ allele (c.296C>T). Discovery of this unique haplotype (TTC) in *Bos indicus* breed and crossbreed cattle prescribed that nucleotide divergence in *MC1R* obtained from *Bos indicus* did not provide to variation in the degree of black in EDE+ heterozygotes [45]. The dominant allele ED, owning a particular amino acid substitution from leucine to proline is reported in bovine E locus [14]. Three leucine amino acid residues were changed to leucine-proline-leucine motifs found in mouse ESO allele. In bovine, proline-leucine-leucine read motifs were found in the ED allele region [14]. We also observed the same kind of results in Karan Fries cross-breed cattle having dominant black color due to the amino acid substitution at 99th position. Moreover, we recognized extensive haplotypes (due to linkage) that stretched for more than 1 Mb either side of *MC1R*. Each EDE+ having a couple of distinct alleles from the *MC1R* gene were attributed from the haplotypes of their progenitors to be heterozygous for the CCT/TTC *MC1R* haplotypes. Inside families, heterozygotes that received the related set of long-range haplotypes revealed a broad change in their degree of black coat color pigmentation; consequently, we hypothesize that it is unlikely that a regulative modification associated with *MC1R* could explain the observed black and white patch effect.

Coat color might be a complex trait, which is linked to many genes, pathways, or networks. *MC1R*, a known key determinant of color phenotype in bovine [46]. The transmembrane domain is composed of several polar and non-polar amino acid changes in the transmembrane domain have been described to regulate gene function due to variation of the protein localization and interaction with different molecules [47, 48]. Due to SNP changes (c.844C>A, p281T>N) might alter the function and the structure of *MC1R* protein. A novel Single SNP in Karan Fries cattle (black and white color) breed compared to indigenous Sahiwal cattle (reddish yellow color) due to amino acid changes threonine to asparagine in A 7-TM inner region of the *MC1R* gene. Because of the protein sequence changes due to the hydroxylation of the amino acid threonine which will involve in the pathway of the pheomelanin synthesis it can cause reddish coat color. Due to the lack of the eumelanin synthesis in the production of black and white color. Several studies reported that in mammals, the pigmentation of the coat color can be affected by 150 to 300 genes reported by [49, 50]. Various coat color phenotypes distinguished by the Reggiana and Holstein cattle are recognized in the MC1R gene region, on the eighteenth chromosome. These two breeds showed the intense allele frequency divergence in the *MC1R* region was observed by [51], which recently reported that the *MC1R* gene (c.871G>A) SNP can cause the mutation in brown Japanese cattle [52]. Senczuk et al. reported that different grey and non-grey coat color cattle breeds [53]. The *MC1R* gene variant is associated with black color in Swiss Holstein cattle breed [54]. Recently, Kasprzak et al. described *MC1R* polymorphism in different coat color cattle breeds of Central Europe [55].

## Conclusions

Analysis of Sahiwal MC1R gene revealed that the SNP c.844C>A or p.281 T>N might be the possible reason for its coat color variation from Karan Fries cattle. Further molecular studies need to be conducted to prove this association with the synthesis of different melanins.

## Supplementary Information

**Additional file 1: Table S1.** Web tools and function for bioinformatics analysis. **Figure S1.** Hydrophilicity and hydrophobicity analyses. **Figure S2.** Signal peptide prediction. **Figure S3.** Transmembrane domain prediction. **Figure S4.** Phosphorylation site prediction. **Figure S5.** Secondary structure prediction.

## Data Availability

GenBank sequence files are available from the NCBI database (Accession ID: MG373575 to MG373605) other supporting data is provided in the supplementary material.

## Abbreviations

*MC1R*: Melanocortin 1 receptor gene
SNPs: Single nucleotide polymorphisms
7-TM: Seven transmembrane domains
GPCR: G-protein coupled receptor
PCR: Polymerase chain reaction
gDNA: Genomic deoxyribonucleic acid
α-MSH: Alpha melanocyte-stimulating hormone
ORF: Open reading frame
CDS: Coding sequence
