# Supplementary material for: Molecular characterization of coat color gene in Sahiwal versus Karan Fries bovine
Source: J Genet Eng Biotechnol. 2021 Jan 29;19:22. doi: 10.1186/s43141-021-00117-2 (PMC7846656; doi:10.1186/s43141-021-00117-2)
Supplement: Supplementary file 1 — Additional file 1: Table S1. Web tools and function for bioinformatics analysis. Figure S1. Hydrophilicity and hydrophobicity analyses. Figure S2. Signal peptide prediction. Figure S3. Transmembrane domain prediction. Figure S4. Phosphorylation site prediction. Figure S5. Secondary structure prediction. [file 43141_2021_117_MOESM1_ESM.docx]

**Supplementary Table 1** Web tools and function for bioinformatics analysis

| **Bioinformatics analysis Web tools** | **purpose** |
| --- | --- |
| **ProtParam**  (https://web.expasy.org/cgi-bin/protparam/protparam) | Physico and chemical analysis |
| **Protscale**  (https://web.expasy.org/protscale/) | Hydrophilic and hydrophobic analysis |
| **ORF Finder**  (http://www.ncbi.nlm.nih.gov/orffinder/) | Open reading frame prediction |
| **SignalP 4.0 Server**  (http://www.cbs.dtu.dk/services/SignalP/) | Signal peptide prediction |
| **TMHMM Sever v.2.0**  (http://www.cbs.dtu.dk/services/TMHMM/) | Transmembrane domain prediction |
| **NetPhos2.0 Server**  (http://www.cbs.dtu.dk/services/NetPhos/) | Phosphorylated site prediction |
| **NetSurfP-2.0 Server**  (http://www.cbs.dtu.dk/services/NetSurfP) | Secondary structure prediction |
| **PROTTER**  (http://wlab.ethz.ch/protter) | Integrated membrane proteins Predictions |
| **Ensemble**  https://asia.ensembl.org/index.html | Genomics and Proteomics analysis |


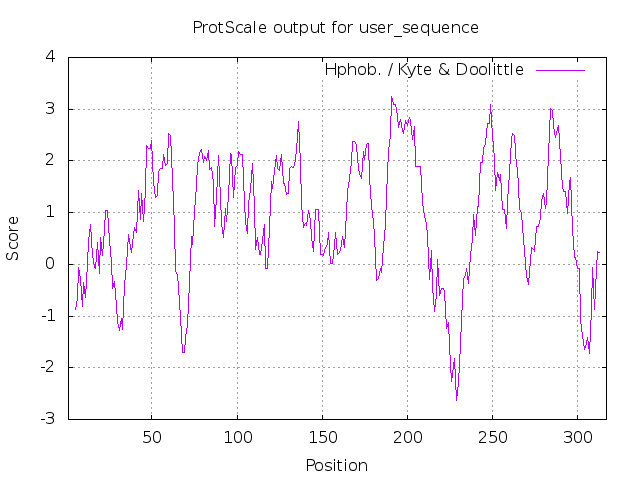


**Supplementary Fig. 1** Hydrophilicity and hydrophobicity analyses


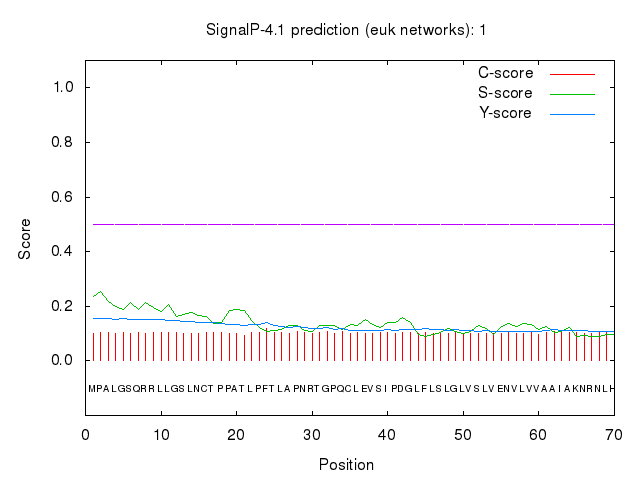


**Supplementary Fig. 2** Signal peptide prediction


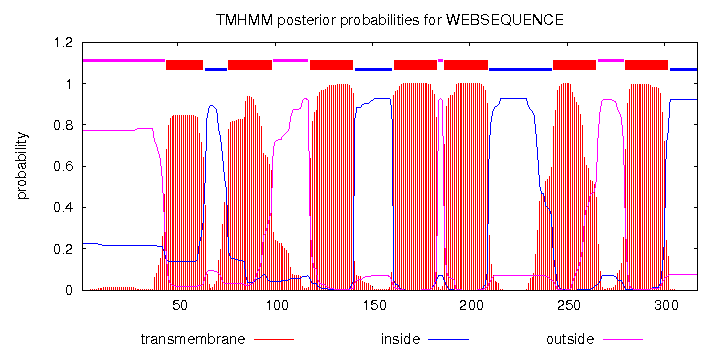


**Supplementary Fig. 3** Transmembrane domain prediction


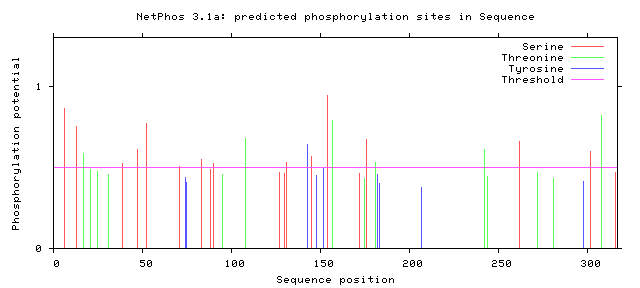


**Supplementary Fig. 4** Phosphorylation site prediction

**
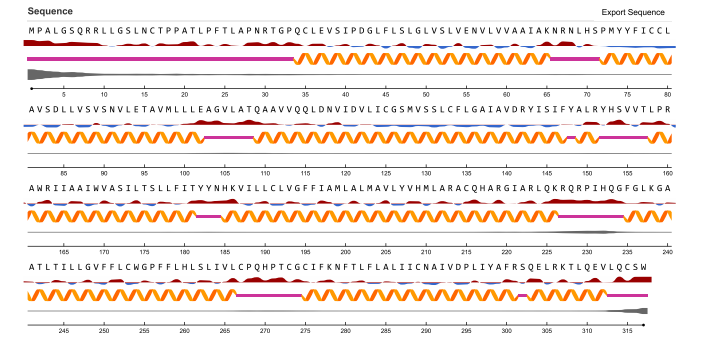
**

**
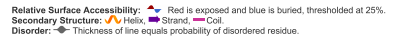
**

**Supplementary Fig. 5** Secondary structure prediction
